# Preparation and characterization of form-stable phase change material/end-of-life tires composites for thermal energy storage

**DOI:** 10.3906/kim-1911-23

**Published:** 2020-04-01

**Authors:** Yeliz KONUKLU

**Affiliations:** 1 Nanotechnology Application and Research Centre, Niğde Ömer Halisdemir University Turkey; 2 Department of Chemistry, Niğde Ömer Halisdemir University Turkey

**Keywords:** Phase change material, end-of-life tires, thermal energy storage, composite material

## Abstract

The management of end-of-life tires (ELT) waste gains importance in aspect of possible environmental and economic issues so the waste recycling becomes unavoidable. This study describes the fabrication and characterization of a new phase changing material (PCM)/ELT microcomposites that could be used in thermal energy storage. Paraffin together with the 4 fatty acids and ELT rubber powder are used as PCMs and as the supporting material, respectively. Paraffin/ELT composites are fabricated, as well, by the vacuum impregnation method in order to investigate the effect of the preparation method. The thermal, morphological, and chemical properties of the prepared PCM/ELT rubber microcomposites are determined with differential scanning calorimetry (DSC), scanning electron microscopy (SEM), and FTIR, respectively. Additionally, the effects of the PCM amount on the composite materials are investigated. As a result of DSC results, the melting temperature and latent heat of the paraffin/ELT rubber microcomposites are determined as 37.2 °C and 80.79 J/g for direct impregnation method and 36.8 °C and 80.69 J/g for vacuum impregnation method, respectively. Based on the findings of this study, it can be claimed that PCM/ELT rubber microcomposites can be used as energy-saving materials in thermal energy storage applications.

## 1. Introduction

The world’s energy needs are increasing daily, making energy conservation more vital in many sectors such as technological innovations and increment of living standards. The outstanding issue in this regard is that energy conservation would be considered at the level of energy savings and up to the level of energy production. Therefore, creating awareness regarding energy conservation becomes remarkable. In the last few years, many heating and cooling applications have benefitted from using thermal energy storage in PCMs [1–2] because of PCMs’ isothermal nature and high volumetric capacity.

Waste is one of the most common global environmental problems. In recent years, many researches have attracted attention on developing biodegradable materials in order to reduce wastes and protect the environment [3–4]. In addition to producing biodegradable materials, the recycling of nonbiodegradable materials has gained importance as well in waste management. For instance, waste tires have been recently considered a threat for environment since they are counted as nonbiodegradable [5]. An estimated 1 billion tires worldwide (approximately 17 million tons) complete their lives every year so a huge amount of waste arises in turn that recycling is really needed for both economy and environment [6]. Thus, new methods for evaluating and recycling these waste products should be investigated. End-of-life tires (ELTs) are very resistant and durable as they could be useful in a variety of products [7]. ELTs are mainly composed of natural or synthetic rubber, carbon black, steel, fibres, and several components. ELT rubber is obtained by separation of ELT from textile fibres and steel, and then granulated to obtain the granular or powder form. It can be used as a sustainable raw material in new rubber composites and it is one of the effective solutions of ELTs management [8].

Many researches have focused on reuse of powdered or granulated ELT rubber in some applications such as coatings for electrical cables, binders for porous asphalt automobile parts, coatings, shoe soles, sports flooring and facilities, floor tiles, livestock mattresses, roofing materials, crash cushions, dyes, inks, solid wheels [9] and additionally construction applications as cement-based and insulation composite materials [10]. Furthermore, ELT rubber powder is used as matrix in rice husk/waste tire rubber composites [11]. Diaconescu et al. have reported a significant improvement in tensile strength when compared to the Portland cement concrete when they use polymer concrete, which was prepared by tire powder composites [12].

PCMs are currently used in building materials, photovoltaic elements, temperature-sensitive electronic devices, preparing temperature-controlled textile fabrics, and temperature-controlled packaging design for the purpose of reducing the heating and/or cooling load of applications. The liquefaction of PCMs during the phase changes and their leakage from the structure restrict their application areas. Recently, there have been many studies on preparing composites of phase-change materials (PCMs) in order to prevent leakage during phase changes [13–15]. Encapsulation can be proposed as novel yet practical solution to overcome the mentioned issue, where another possible solution is to prepare composites of PCMs with inorganic or organic supporting materials [16] similar to encapsulation [17]. Several PCM composites have been described in the literature so far. For example, myristic acid (MA)/high-density polyethylene (HDPE)/NaO and NG composites are prepared by Tang et al. in order to increase the final product’s thermal conductivity [18]. The authors report that the composites can be prepared with 70% MA and 12% nano–powder in the HDPE matrices with an increment of 95%–121% in thermal conductivity. In one of our previous studies, we were successfully synthesized paraffin/xonotlite composites with a melting point of 35.05 °C and a relatively high heat of fusion of 65.8 kJ/kg [19]. Konuklu et al. fabricated sepiolite-based PCM nanocomposites with melting phase change enthalpies of 62.08 J/g for paraffin and 35.69 J/g for decanoic acid composites [20]. In another study, porous diatomite was used as the supporting matrix by aiming the protection of PCM during the phase change process. It was shown as a result that the obtained composite exhibited good thermal reliability without any leakage [21]. Guo et al. prepared composite materials by incorporating diatomite-stabilized paraffin as a thermal energy storage material [22]. Mitran et al. prepared lauric acid and mesocellular foam silica using a solvent-less method [23].

To the best of our knowledge, our study is one of the first example in this research field regarding the preparation of PCM/ELT rubber microcomposite materials with the capacity to store thermal energy. The aim of this study is to prepare PCM/ELT rubber composite materials that could be used as energy-saving materials in building applications. To this end, 5 series of PCM/ELT rubber composites have been fabricated and characterized.

## 2. Materials and methods

### 2.1. Materials

**Figure 1:**
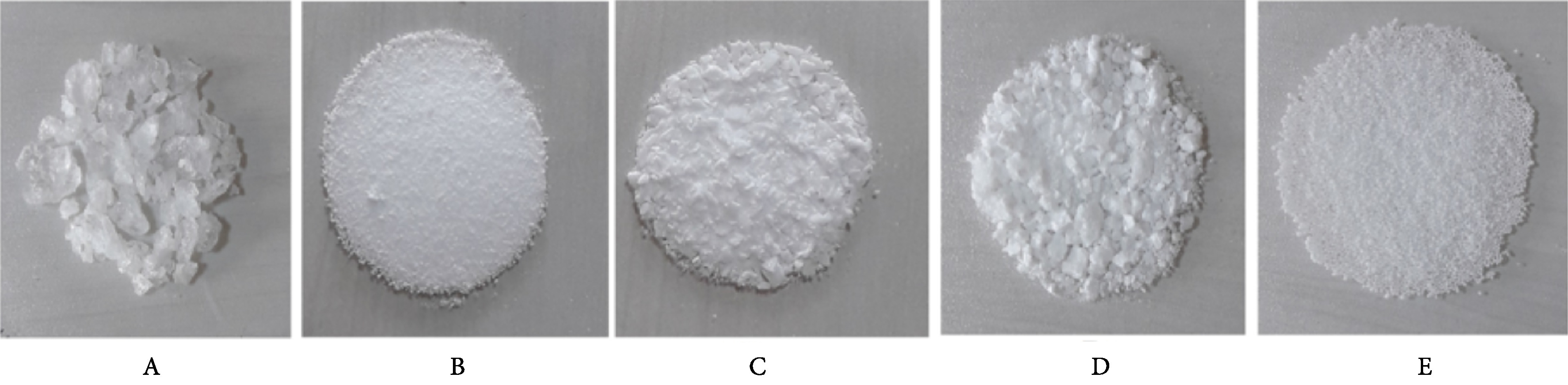
PCMs used in this study (A: paraffin, B: palmitic acid, C: myristic acid, D: lauric acid, E: stearic acid.

Paraffin (P), palmitic acid (PA), and stearic acid (SA) were acquired from Merck Chemical, Aldrich, and TEKKIM, respectively, while amyristic acid (MA) and lauric acid were obtained (LA) from Acros Organics. Molecular and structural formulas of fatty acids are summarized in Table 1. Figure 1 shows chemical structures of the fatty acids and image of the PCMs used in this study. Epoxy and epoxy hardener behave as the auxiliary.

**Table 1:** Chemical structures of the fatty acids used in this study.

Sample	Molecular formula	Structural formula
Palmitic acid	C16H32O2	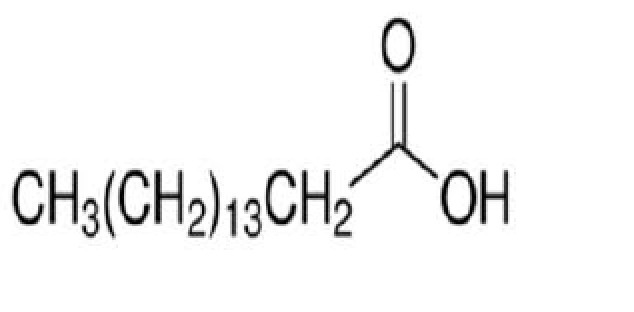
Myristic acid	C14H28O2	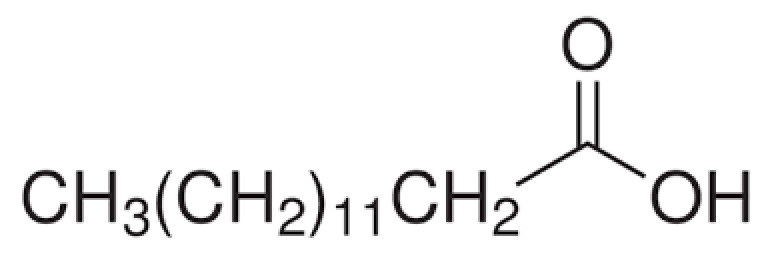
Lauric acid	C12H24O2	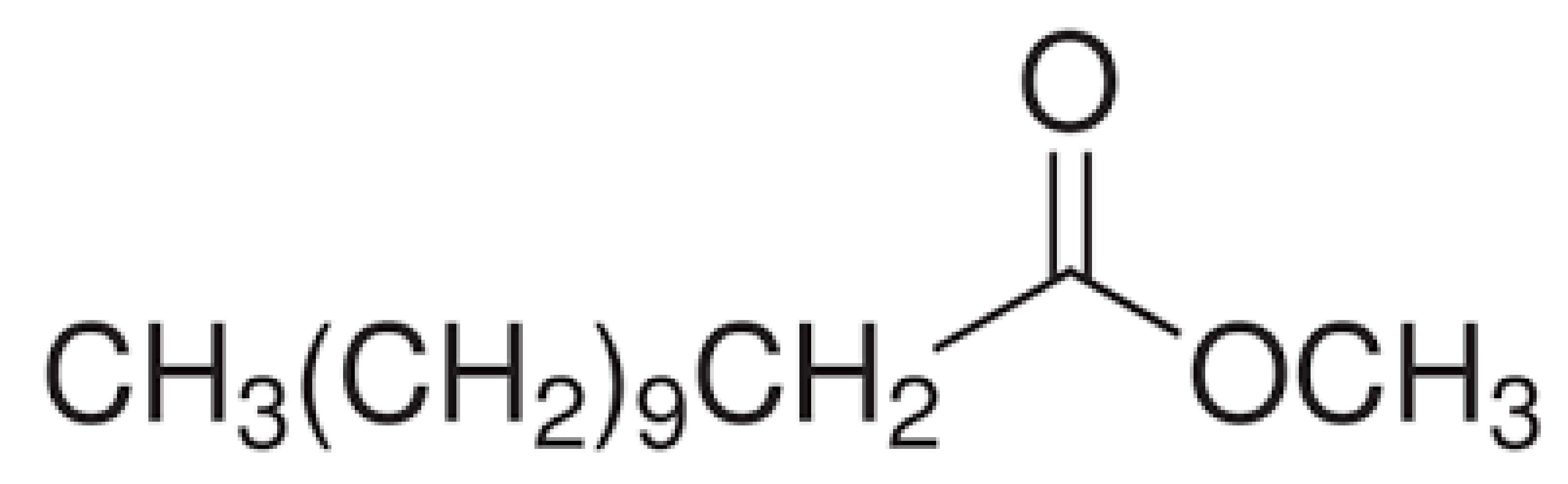
Stearic acid	C18H36O2	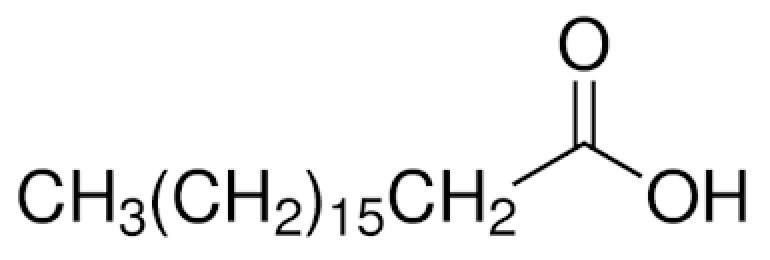

The ELT rubber powder (also can be called as tire rubber powder, rubber powder or regenerated tire rubber) provided from UN-SAL (Turkey) was used as a composite matrix. The properties of the ELT rubber powder sample as indicated in the certificate analysis of the products are presented in Table 2.

**Table 2:** Properties of used ELT rubber powder*.

Properties	
Total polymer content (natural and synthetic rubbers)	58% minimum
Acetone extract:	5%–20 %
Carbon black:	25%–35 %
Ash at 550 °C	15% maximum
Sulphur:	1%–3%
Colour	Black

*Data from certificate of analyses.

**Figure 2:**
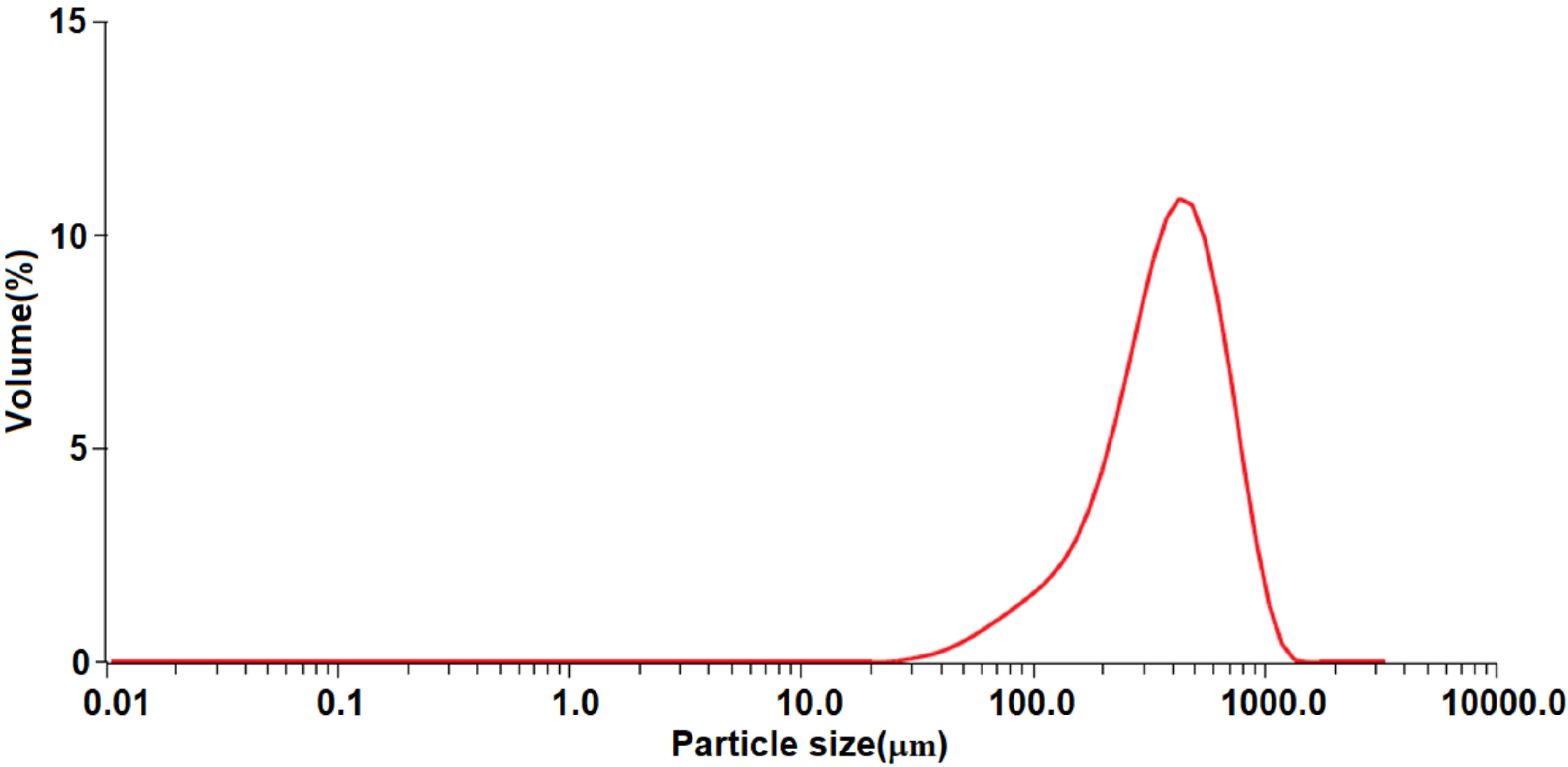
Particle size analyses of ELT rubber.

The particle size distribution analyses of ELT rubber powder used in this study were determined by laser diffraction particle size analyser (Malvern-Mastersizer 3000). According to the particle size distribution analyses presented in Figure 2, the ELT rubber powder has a median grain size (d50) of 381μm and d90 value of 717μm.

### 2.2. Preparation and characterization of samples

During the PCM/ELT rubber microcomposite preparation process, all materials were used without any purification. The PCM/ELT rubber microcomposites were prepared as described in detail in our patent application [24]. In the direct impregnation method, the PCMs were heated to a temperature above their melting points. In the second stage, ELT rubber in a powder form were slowly added to the molten PCMs and stirring was kept at 100 rpm. After composite preparation, a small amount of the resin was added in order to provide a proper shape. The shaped composites afterward were left at room temperature for 24 h to dry. As presented in Table 3, 15 PCM/ELT rubber microcomposites were prepared using 5 different PCMs.

**Table 3:** PCM content of PCM/ELT rubber microcomposites.

Sample	PCM	PCM amount %
mcA	P	44
mcA1	P	48
mcA2	P	52
mcB	PA	44
mcB1	PA	48
mcB2	PA	52
mcC	MA	44
mcC1	MA	48
mcC2	MA	52
mcD	LA	44
mcD1	LA	48
mcD2	LA	52
mcE	SA	44
mcE1	SA	48
mcE2	SA	52

To investigate the effect of the preparation method, paraffin composites were also fabricated using the vacuum impregnation method. In the vacuum impregnation method, a laboratory-type vacuum reactor was used to prepare the PCM/ELT rubber microcomposites. The temperature of the vacuum reactor was controlled by a magnetic heater or hot water bath. The goal of this technique was to evacuate the air in the environment by the help of vacuum and thereby increase the ratio of absorbed PCM in the composite.

The thermal properties of the PCMs and PCM/ELT rubber microcomposites were investigated by a DSC (Perkin Elmer) at a heating and cooling rate of 5 °C/min. The analyses took place between 25 °C and 70 °C. The composite’s PCM content was determined using the Eq. (1).

PCM% = (ΔHcomposite ÷ ΔHPCM) ×100 Eq.(1)

The FTIR analyses of paraffin and the PCM/ELT rubber microcomposites were carried out using a FTIR spectrometer (Perkin Elmer). The morphology of the microcomposites was examined by SEM.

## 3. Results and discussion

### 3.1. Thermal properties

**Figure 3:**
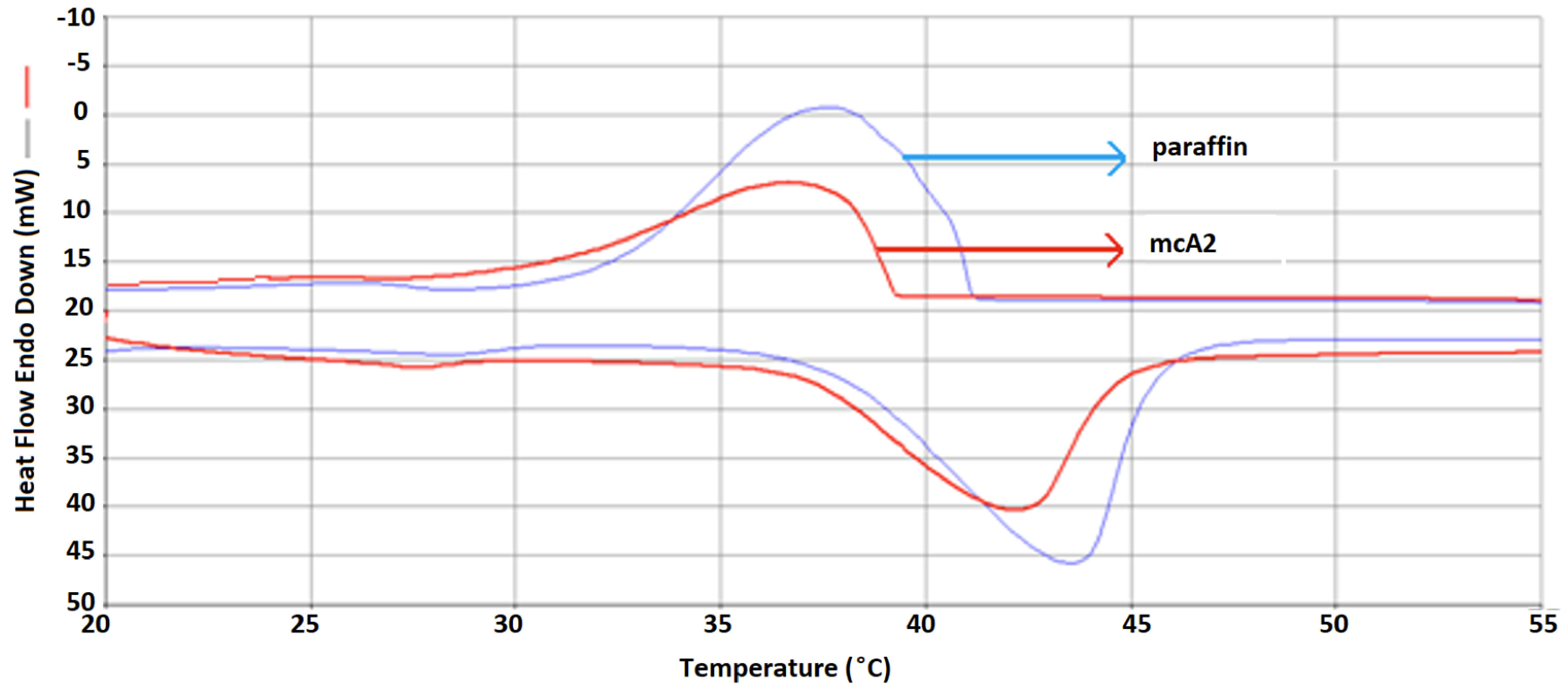
DSC analysis of paraffin and paraffin/ELT microcomposites (mcA2).

The thermal properties of paraffin and PCM/ELT rubber microcomposite prepared with impregnation method determined by DSC analysis are summarized in Table 4. The DSC analysis of paraffin and mcA2 is presented in Figure 3. To obtain the maximally efficient energy storage capacity, the PCM ratio of the composites was increased. Thereby, the melting enthalpy (Hm) and temperature (Tm) of the paraffin was calculated as 134.77 J/g and 37.57 °C, respectively. Additionally, the percentages of paraffin in mcA, mcA1, and mcA2 were found as 30%, 45%, and 60% and the melting enthalpies were 40.47 J/g, 60.92 J/g, and 80.79 J/g, respectively. As can be clearly revealed from the results, the PCM ratios in the paraffin/ELTp composites directly affected the energy storage efficiencies of composites. Moreover, it can be concluded from the DSC results that the obtained mcA2 composition had 80.79 J/g melting enthalpy and –76.39 J/g freezing enthalpy when the weight ratio of paraffin in the composite was 52%. These values indicate that the paraffin content in the composite was 60%, i.e. although the paraffin content in the composite was 52%, a yield of 60% in the composite was obtained. Furthermore, it has been thoroughly reported in literature that the interaction between paraffin and carbon-based materials in the composite has a positive or negative influence on latent heat [25]. It can be concluded that the interaction paraffin and ELT rubber has a positive influence on enthalpy of paraffin/ELT rubber composites.

**Table 4:** Thermal properties of paraffin/ELT rubber microcomposites.

Sample	T_om_ (°C)	T_pm_ (°C)	T_em_ (°C)	H_m_ (J g−1)	T_oc_ (°C)	T_pc_ (°C)	T_ec_ (°C)	H_ec_ (J g−1)	PCM%
paraffin	37.57	43.47	45.43	134.77	41.14	37.63	32.18	–137.61	100
mcA	36.32	41.22	42.95	40.47	38.73	37.25	30.95	–41.53	30
mcA1	39.69	41.66	44.07	60.92	39.02	37.10	30.77	–52.29	45
mcA2	37.20	42.25	44.51	80.79	39.28	36.83	30.58	–76.39	60

T_om_: Onset melting temperature of DSC curve. T_pm_: Melting peak temperature of DSC curve. T_em_: Endset melting temperature of DSC curve. H_m_: Melting enthalpy of PCMs in DSC curve. T_oc_: Onset crystallizing temperature of DSC curve. T_pc_: Crystallizing peak temperature of DSC curve. T_ec_: Endset crystallizing temperature of DSC curve. H_c_: Crystallization enthalpy of PCMs in DSC curve

Regarding the palmitic acid/ELT rubber microcomposites, the mcB, mcB1, and mcB2 microcomposites were prepared by increasing the palmitic acid ratio. The latent heat and Tm values of the palmitic acid were 211.51 J/g and 60.81°C, respectively as shown in table 5. The percentages of palmitic acid were 23%, 26%, and 38% and the phase change enthalpies were 49.56, 56.46, and 82.25 J/g in mcB, mcB1, and mcB2, respectively. Similar to the results for the paraffin/ ELT rubber microcomposites, the phase change enthalpy also increased as the amount of PCM increased. The maximum yield (38%) was obtained with the mcB2 composition. As a result, it can be concluded that the amount of PCM used in the composite was a major influence on the final product’s energy storage capacity.

**Table 5:** Thermal properties of palmitic acid/ELT rubber microcomposites.

Sample	T_om_ (°C)	T_pm_ (°C)	T_em_ (°C)	H_m_ (J g−1)	T_oc_ (°C)	T_pc_ (°C)	T_ec_ (°C)	H_ec_ (J g−1)	PCM%
Palmitic	60.81	64.57	67.15	211.51	59.22	57.00	59.22	–218.36	100
mcB	49.14	60.30	62.25	49.56	55.49	54.69	50.12	–54.99	23
mcB1	48.00	54.97	66.87	56.46	53.60	50.98	41.58	–24.18	26
mcB2	55.02	62.02	64.06	82.25	56.85	56.08	52.99	–80.58	38

The myristic acid/ELT rubber microcomposites, mcC, mcC1, and mcC2, were prepared by using different myristic acid:ELT ratios meanwhile the amount of resin used in the composites was kept constant. It is presented in table 6 that the latent heat of the myristic acid was 200.25 J/g and the Tm was 53.87 °C. The percentages of myristic acid in mcC, mcC1, and mcC2 were 14%, 37%, and 40% and the phase change enthalpies were 27.84, 74.24, and 81.66 J/g, respectively. The maximum energy storage capacity was obtained with the mcC2 composition. Namely, it would be safe to say that the mcC2 composite is suitable for thermal energy storage (TES) systems.

**Table 6:** Thermal properties of myristic acid/ELT rubber microcomposites.

Sample	T_om_ (°C)	T_pm_ (°C)	T_em_ (°C)	H_m_ (J g−1)	T_oc_ (°C)	T_pc_ (°C)	T_ec_ (°C)	H_ec_ (J g−1)	PCM%
Myristic	53.87	58.60	63.09	200.25	50.77	47.90	44.23	–210.46	100
mcC	37.18	41.97	44.54	27.84	36.45	33.67	27.02	–30.73	14
mcC1	43.90	52.24	54.69	74.24	47.98	44.54	36.06	–54.58	37
mcC2	40.48	47.59	54.53	81.66	46.69	35.36	31.10	–67.01	40

**Figure 4:**
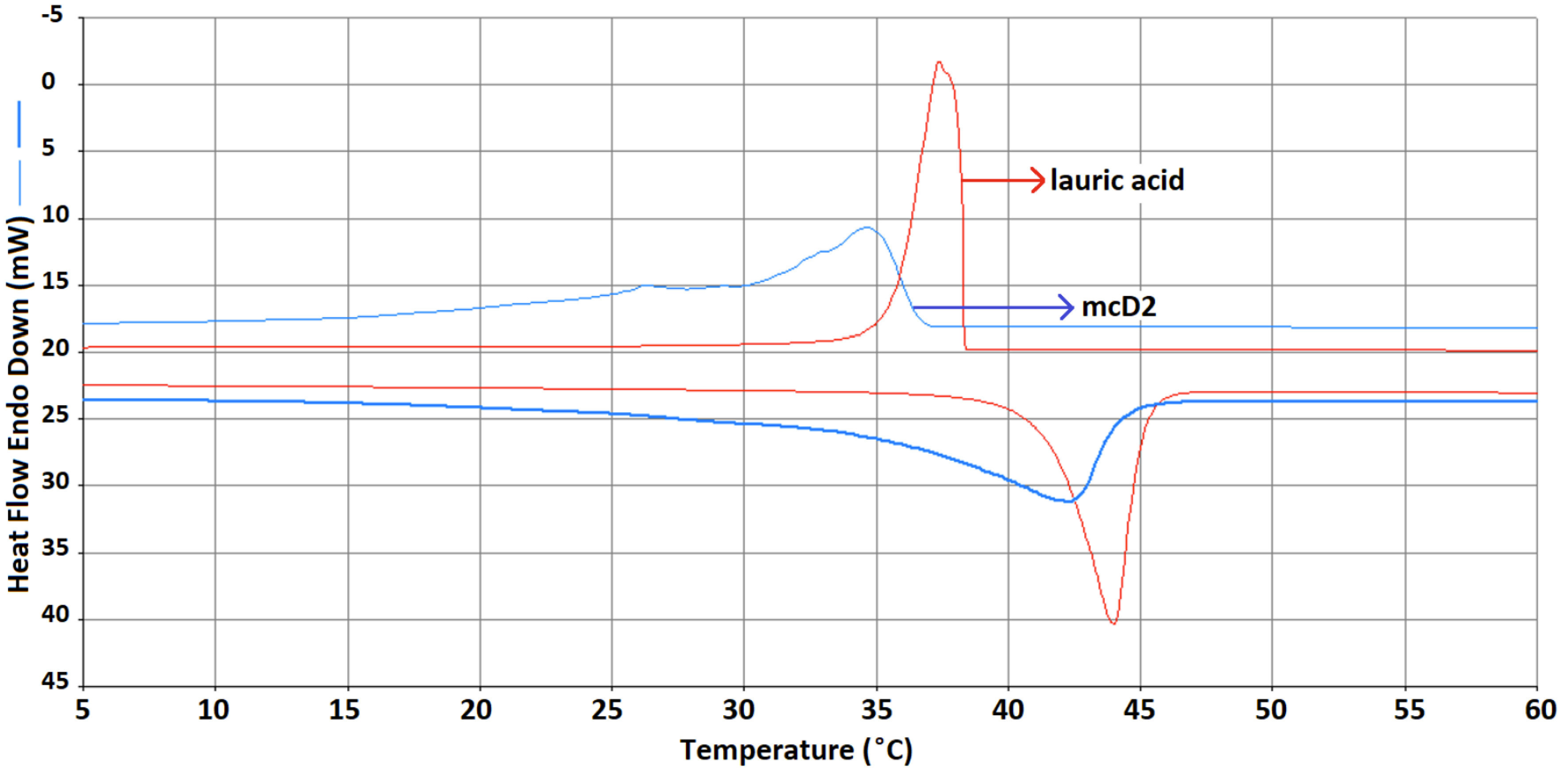
DSC analysis of paraffin and lauric acid/ELT microcomposites (mcD2).

Lauric acid with acceptable melting and freezing temperatures and enthalpies are proper materials for thermal energy storage. The DSC analysis of lauric acid and mcD2 is presented in Figure 4. According to the DSC results presented in Table 7, the percentages of lauric acid in mcD, mcD1, and mcD2 were 14%, 21%, and 31% and the phase change enthalpies were 28.72 J/g, 42.89 J/g, and 62.15 J/g, respectively. Specifically, the analysis results of mcD2 (which had an energy efficiency value of 31%) revealed the melting and freezing temperatures and enthalpies as 34.00 °C, 36.64 °C, and 62.15 J/g, –61.36 J/g, respectively. The lauric acid contained in the composite displayed supercooling properties, i.e. the melting and freezing temperatures were reduced by about 10 °C. It is estimated that supercooling occurs when the resin is added to the composite. Although the composites showed supercooling properties, they still have acceptable thermal properties for thermal energy storage applications.

**Table 7:** Thermal properties of lauric acid/ELT rubber microcomposites.

Sample	T_om_ (°C)	T_pm_ (°C)	T_em_ (°C)	H_m_ (J g−1)	T_oc_ (°C)	T_pc_ (°C)	T_ec_ (°C)	H_ec_ (J g−1)	PCM%
Lauric	43.66	48.54	52.60	196.61	40.98	37.02	33.11	–205.19	100
mcD	24.11	32.68	40.31	28.72	20.18	15.16	9.89	–31.75	14
mcD1	30.18	34.79	40.54	42.89	28.78	23.84	19.32	–41.57	21
mcD2	34.00	42.24	44.17	62.15	36.64	34.58	30.23	–61.36	31

To obtain the stearic acid/ELT rubber microcomposites, the mcE, mcE1, and mcE2 microcomposites were prepared. According to the DSC results (Table 8), the stearic acid content in mcE, mcE1, and mcE2 was 9%, 28%, and 40%, respectively. As can be seen in the table, the melting and freezing temperatures and phase change enthalpies were determined as 67.90 °C and 202.64 J/g for stearic acid 48.17 °C and 82.12 J/g for mcE2, respectively. The DSC results show that stearic acid in the composite show supercooling properties similar to lauric acid.

**Table 8:** Thermal properties of stearic acid/ELT rubber microcomposites.

Sample	T_om_ (°C)	T_pm_ (°C)	T_em_ (°C)	H_m_ (J g−1)	T_oc_ (°C)	T_pc_ (°C)	T_ec_ (°C)	H_ec_ (J g−1)	PCM%
Stearic	67.90	59.68	77.34	202.64	64.84	61.80	56.84	–215.72	100
mcE	58.43	60.69	65.13	18.94	56.86	52.60	47.86	–15.93	9
mcE1	42.27	54.47	57.38	57.74	47.97	40.98	34.29	–40.95	28
mcE2	48.17	55.58	57.63	82.12	50.85	49.72	45.93	–51.17	40

The most appropriate composite among all materials was identified as the paraffin/ELT rubber microcomposites due to its supercooling property together with the maximum efficiency. In order to investigate the effect of the preparation method, the paraffin composites of mcA*, mcA1*, and mcA2* were also prepared using the vacuum impregnation method. mcA*, mcA1*, and mcA2* were prepared with the same recipe of mcA, mcA1, and mcA2, respectively. The DSC results of composites are summarized in Table 9. According to the DSC results, the percentages of paraffin in mcA*, mcA1*, and mcA2* were 45%, 51%, and 60 % and the phase change enthalpies were 61.35, 68.96, and 80.69 J/g, respectively. Consequently, mcA2* has the maximum latent heat storage capacity in comparison with mcA* and mcA1*.

**Table 9:** Thermal properties of paraffin/ELT rubber microcomposites (vacuum impregnation method).

Sample	T_om_ (°C)	T_pm_ (°C)	T_em_ (°C)	H_m_ (J g−1)	T_oc_ (°C)	T_pc_ (°C)	T_ec_ (°C)	H_ec_ (J g−1)	PCM%
paraffin	37.57	43.47	45.43	134.77	41.14	37.63	32.18	–137.61	100
mcA*	36.93	43.01	45.94	61.35	39.10	35.45	29.06	–55.25	45
mcA1*	36.83	42.12	44.67	68.96	39.27	36.30	29.84	–63.82	51
mcA2*	36.83	41.97	44.63	80.69	39.24	36.63	29.94	–73.39	59.9

*with vacuum impregnation.

The results indicate that the vacuum impregnation method provides better phase change enthalpies in mcA* and mcA1* rather than mcA and mcA1 prepared with impregnation method. As can be expected, the air contained in the ELT powder was evacuated by vacuum impregnation method and further absorption of paraffin was achieved. However, the composite preparation method has no significant effect on thermal properties when the PCM content reached to 52% in the PCM/ELT rubber microcomposites

**Figure 5:**
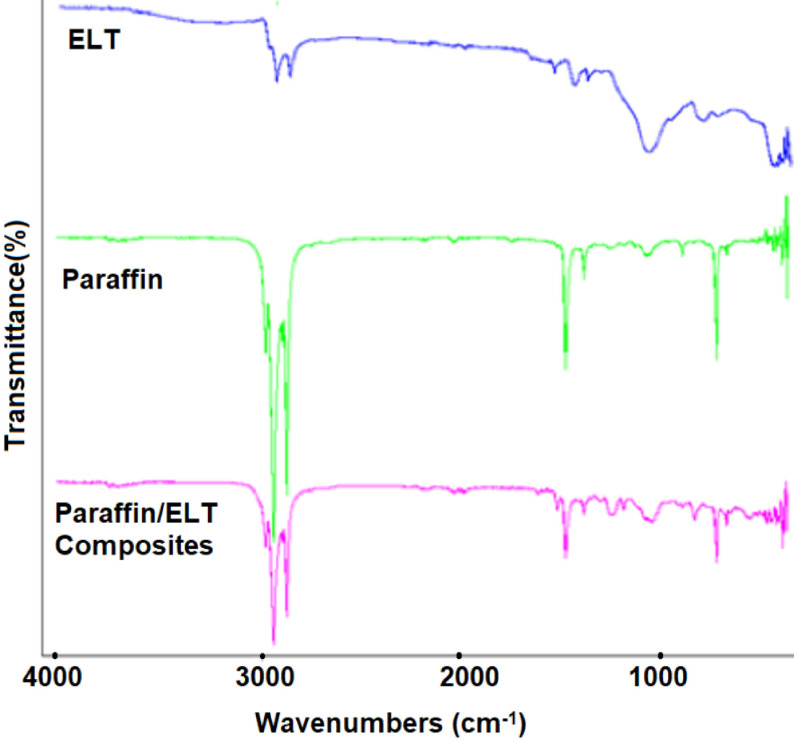
FTIR analyses of ELT, paraffin, and paraffin/ELT microomposites.

**Figure 6:**
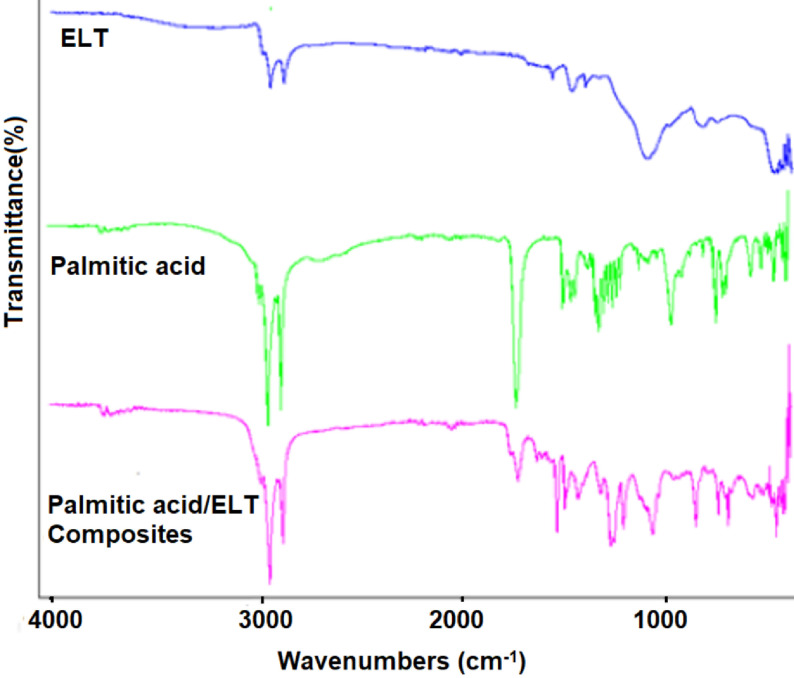
FTIR analyses of ELT, palmitic acid, and palmitic acid/ELT microcomposites.

**Figure 7:**
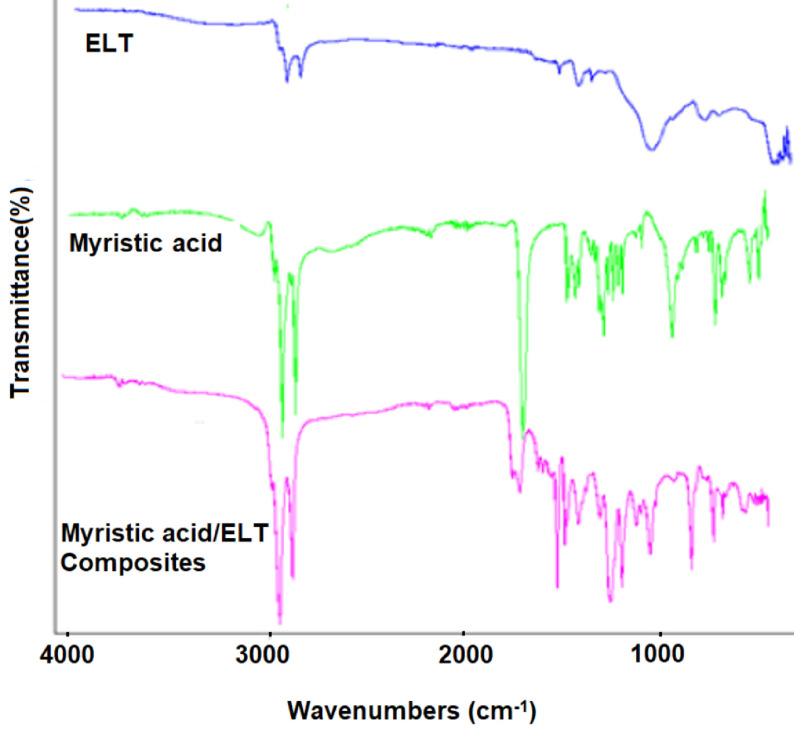
FTIR analyses of ELT, myristic acid, and myristic acid/ELT microcomposites.

FTIR analyses were performed for the chemical analysis of the PCM/ELT rubber microcomposites to identify whether the chemical structure of the PCM has changed during the composite preparation process. The spectra obtained as a result of the FTIR analysis are given in Figures 5–7. In the ELT spectra, some specific absorption areas were observed in the range of approximately 2800–3000 cm−1 for C-H stretching vibration, and 1350–1500 cm−1 for CH2 deformation similar to any previous report [26]. Moreover, the FTIR spectra of paraffin, palmitic acid and myristic acid demonstrate a similarity with the literature [27–29]. The peaks at approximately 2950 cm−1 , 2900 cm−1 , and 2800 cm−1 as shown in Figures 3–5 were attributed to the symmetric and asymmetric stretching vibration of –CH2 and –CH3 groups of paraffin and fatty acids. Paraffin has specific peaks at 1450 cm−1 and 750 cm−1 while myristic and palmitic acids have –COOH stretching vibration peaks at approximately 1700 cm−1 . As can be concluded from the FTIR analysis, there are specific peaks for both the PCMs and ELT in the spectra of the prepared composites. FTIR analysis confirms that the composite preparation process was successfully accomplished.

### 3.2. Morphology and microstructure

**Figure 8:**
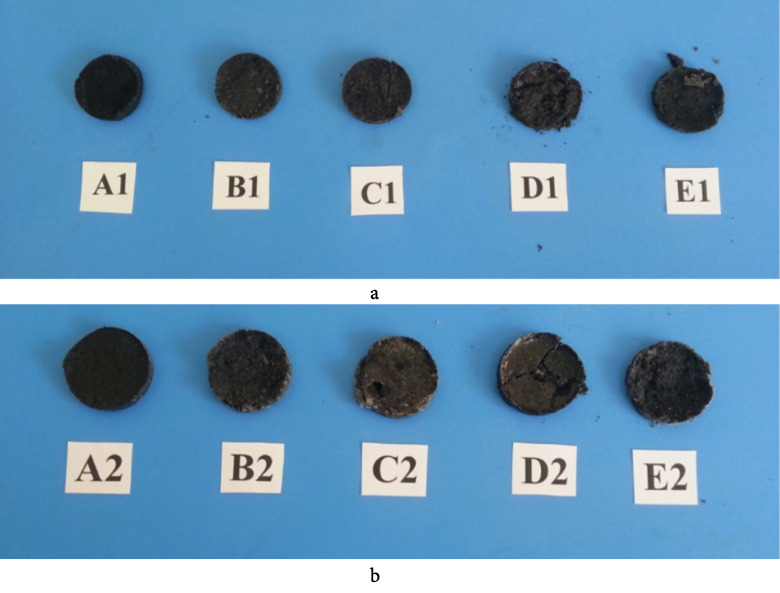
PCM/ELT microcomposites.

The prepared PCM/ELT rubber composites have similar structural properties in the room temperature conditions as presented in Figure 8. It is clear that paraffin, palmitic acid and myristic acid have more stable surface (without cracks) than LA and SA composites.

**Figure 9:**
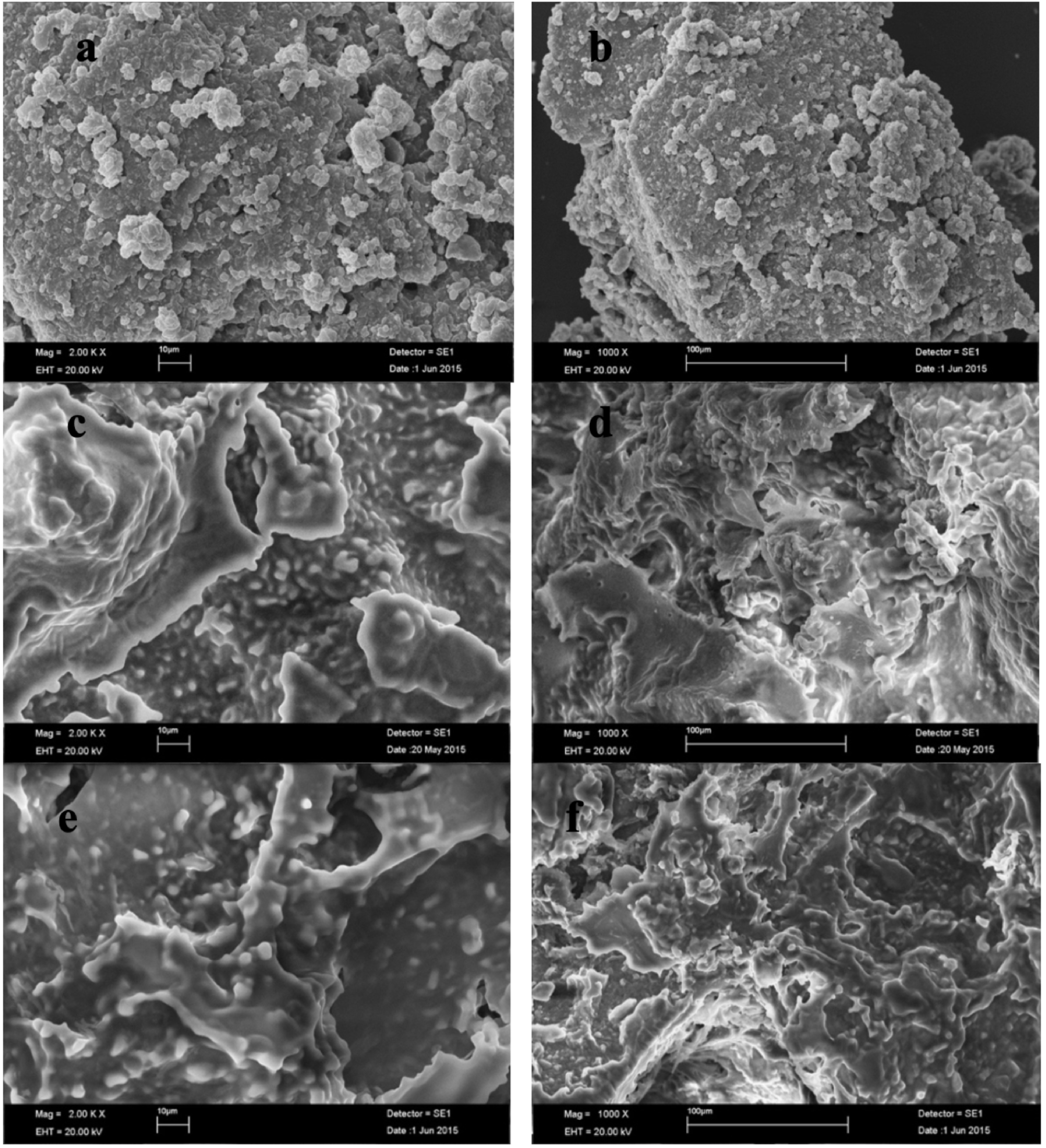
SEM graphs of (a-b) ELTp, (c-d) parafin/ELTp composites, (e-f) parafin/ELTp composites (with vacuum impregnation method).

The morphology analysis of untreated ELT rubber and paraffin/ELT rubber microcomposites prepared by the impregnation and vacuum impregnation methods was performed by SEM. The obtained micrographs are presented in Figure 9. Untreated ELT rubber powders show an aggregated and unsmooth structure [30–31]. The SEM micrographs indicate that the structure of ELT rubber is modified by paraffin similar to what was reported for xonotlite/paraffin- and cellulose-based myristic acid composites [32–33]. It was also found that the structural properties of the composites prepared by the vacuum and nonvacuum impregnation methods show similarities and moreover, they become smoother compared to untreated ELT rubber.

## 4. Conclusion

ELTs are important environmental problems and recycling these waste materials is of great importance for our economy and environment in today’s world. ELT can be used as a new potential supporting material for PCM/ELT rubber microcomposites. The composites were prepared with high thermal energy storage capacity to save energy, reduce the amount of waste, and contribute to the economies of the country and the world. Depending on the insights we gained in this study that focused on the microcomposites, we prove that getting consistent results is crucial for the success of composite-generating processes as microencapsulation. According to analysis results, increasing the amount of PCM led to an increase in the latent heat storage capacity. Therefore, we obtained the maximum composite efficiencies of 60%, 38%, 40%, 31%, 40 for paraffin, PA MA, LA, and SA, respectively.

It was also confirmed that the paraffin/ELT rubber composites were synthesized successfully with a Tm of 37.2 °C and latent heat storage capacity of 80.79 J/g. The effect of composite preparation method on composite efficiency was determined. It can be concluded that in the preparation of paraffin/ELT rubber composites, the choice of method has significant effect when the paraffin content is not higher than 50%. FTIR and SEM results confirmed that the composite preparation process was successfully accomplished.

In conclusion, we claim that PCM/ELT rubber microcomposites can be used as promising energy-saving materials in thermal energy storage applications as insulation material in buildings, for instance. For further investigation, the developed microcomposites could be prepared at large scale and the effects of heating and cooling loads in test rooms could also be investigated.
